# HIF-1α signaling: Essential roles in tumorigenesis and implications in targeted therapies

**DOI:** 10.1016/j.gendis.2023.02.039

**Published:** 2023-03-30

**Authors:** Yan Zhao, Cheng Xing, Yating Deng, Can Ye, Hongling Peng

**Affiliations:** a Department of Hematology, The Second Xiangya Hospital, Central South University, Changsha, Hunan 410011, China; b Hunan Key Laboratory of Tumor Models and Individualized Medicine, The Second Xiangya Hospital, Central South University, Changsha, Hunan 410011, China; c Hunan Engineering Research Center of Cell Immunotherapy for Hematopoietic Malignancies, The Second Xiangya Hospital, Central South University, Changsha, Hunan 410011, China

**Keywords:** Angiogenesis, Hematological malignancies, Hypoxia-inducible factor-1 alpha (HIF-1α), Immune escape, Targeted therapy

## Abstract

The hypoxic microenvironment is an essential characteristic of most malignant tumors. Notably, hypoxia-inducible factor-1 alpha (HIF-1α) is a key regulatory factor of cellular adaptation to hypoxia, and many critical pathways are correlated with the biological activity of organisms via HIF-1α. In the intra-tumoral hypoxic environment, HIF-1α is highly expressed and contributes to the malignant progression of tumors, which in turn results in a poor prognosis in patients. Recently, it has been indicated that HIF-1α involves in various critical processes of life events and tumor development via regulating the expression of HIF-1α target genes, such as cell proliferation and apoptosis, angiogenesis, glucose metabolism, immune response, therapeutic resistance, *etc*. Apart from solid tumors, accumulating evidence has revealed that HIF-1α is also closely associated with the development and progression of hematological malignancies, such as leukemia, lymphoma, and multiple myeloma. Targeted inhibition of HIF-1α can facilitate an increased sensitivity of patients with malignancies to relevant therapeutic agents. In the review, we elaborated on the basic structure and biological functions of HIF-1α and summarized their current role in various malignancies. It is expected that they will have future potential for targeted therapy.

## Introduction

Recently, it has been identified that hypoxia-inducible factor-1 alpha (HIF-1α) is a major transcriptional regulatory factor that responds to hypoxic environments and modulates the expression of many genes in the organism and is closely associated with the biological behavior of malignant tumors.^1^^,^^2^ Importantly, HIF-1α is commonly highly expressed in tumors under conditions of hypoxia or activation of oncogenic pathways. Further studies revealed that HIF-1α not only participates in reducing the efficacy of radiotherapy, chemotherapy, and targeted therapies but also its target genes are involved in a multitude of pathophysiological mechanisms, such as regulation of angiogenesis, cell proliferation and survival, glucose metabolism, immune escape, and iron metabolism.^2^^,^^3^ Therefore, HIF-1α has been recognized to play an important role in diseases that can generate a hypoxic environment (*e.g.*, malignancies).

In the process of carcinogenesis, normal cells in the organism gradually evolve into malignant lesions, and these lesions can ultimately result in the development of a local malignancy followed by distant metastasis.^4^ For example, malignancy occurs when normal cells acquire a transforming mutation in cancer driver genes through a genetic or sporadic event.^4^ Among them, hematological malignancies are a group of heterogeneous diseases that are related to hematopoietic and lymphoid tissues and are the fourth most prevalent cancer.^5^ The etiology is not yet clear, but most scholars believe that their occurrence is linked to genetics,^6^ environment, chemicals, and infections. Strikingly, the occurrence of malignancies exerts a serious impact on the safety of lives and quality of life for patients, especially leading to a poor prognosis for elderly and frail patients. Currently, their treatment regimens include surgery, radiotherapy, chemotherapy, immunotherapy, hematopoietic stem cell transplantation (HSCT), *etc*.^7^, ^8^, ^9^ However, in a significant proportion of patients, clinical outcomes remain quite poor owing to drug resistance to cancer treatment or relapse of the disease. Consequently, the urgent need to elucidate the pathogenesis with a view to discovering new targets is of high importance.

At present, it is widely recognized that the microenvironment in which solid tumors commonly grow is hypoxia.^10^ Indeed, malignant tumors are a serious threat to human health and the hypoxic microenvironment (HME) is tightly correlated with the malignant progression of tumors (*e.g.*, tumor growth, angiogenesis) with complex regulatory mechanisms. HIF-1α is essential for tumor cells to adapt to the HME. Recent evidence confirms that, with the exception of solid tumors such as breast and liver cancer, HIF-1α is also strongly associated with the development and progression as well as chemotherapy resistance and tumor metastasis of hematological malignancies (*e.g.*, acute myeloid leukemia (AML)).^11^ In this review, we briefly outline the basic structure and biological functions of HIF-1α and discuss the progress of research on HIF-1α in multiple solid tumors and hematological malignancies. Collectively, translational therapy with targeted interventions targeting HIF-1α may provide novel insights for the future precision treatment of malignancies.

### Overview of hypoxia-inducible factors (HIFs)

In a recent study, Semenza et al^12^ unveiled the molecular mechanisms underlying the “discovery of how cells sense and adapt to changes in oxygen”. Subsequently, they were awarded the 2019 Nobel Prize in Physiology or Medicine in recognition of their contribution to a deeper understanding of how oxygen levels affect cellular metabolism and the function of physiology. Further, relevant research on the molecular mechanism has proven to be influential in the treatment of diverse human diseases, including anemia and cancer.^12^ In this regard, HIF-1 is a transcription factor related to the regulation of cellular adaptation to the hypoxic environment identified by Semenza et al^13^ during hypoxia induction in hepatocellular carcinoma (HCC) cell lines.

HIFs, a family of transcription factors, are key regulatory factors involved in the modulation of the cellular response to hypoxic stress.^14^ Among them, it is currently reported that the most important proteins activated in hypoxia are three HIFs, *i.e.*, HIF-1, HIF-2, and HIF-3.^15^ To our knowledge, the first two, HIF-1 and HIF-2, are mainly accountable for the transcription of relevant genes triggered by hypoxia, whereas HIF-3, aside from inducing the expression of target genes, also negatively regulates the activity of HIF-1 and HIF-2, thereby indirectly inhibiting the expression of oncogenes.^16^, ^17^, ^18^

**Figure 1:**
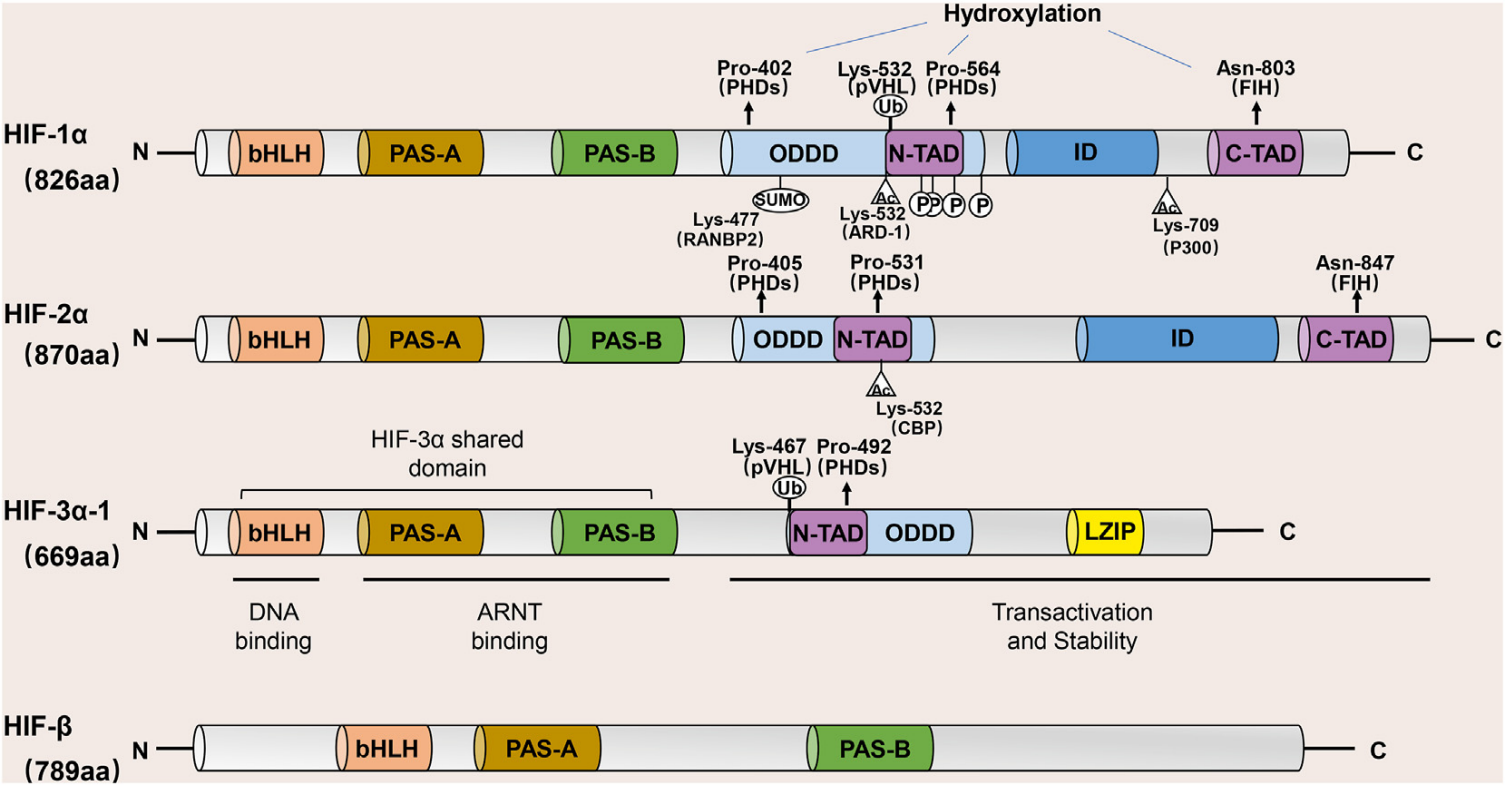
HIF protein domains. Currently, three oxygen-sensitive α-subunit isoforms of HIF have been identified in humans (HIF-1α, -2α, and -3α) and form heterodimers with the oxygen-insensitive β-subunit (also known as the aryl hydrocarbon receptor nuclear translocator (ARNT)). The HIF protein consists of several conserved domains, including a DNA-binding (basic Helix-Loop-Helix, bHLH) domain, a protein/protein interaction and dimerization (PER-ARNT-SIM, PAS-A and PAS-B) domain, an oxygen-dependent degradation domain (ODDD), a transcriptional activation domain (N-TAD, C-TAD), and a repressive domain (ID). ARNT, aryl-hydrocarbon-nuclear receptor translocator; CBP, cyclic adenosine monophosphate response element-binding protein; FIH1, factor-inhibiting HIF-1; HIF-1α, hypoxia-inducible factor-1 alpha; p300, coactivator acetyltransferase; PHD, proline hydroxylase domain; pVHL, von Hippel-Lindau tumor suppressor protein.

All three HIFs are constituted by two subunits, a heterodimer formed by the oxygen-sensitive α-subunit and the oxygen-insensitive β-subunit.^15^ To date, three isoforms of α-subunits for HIF have been identified in humans or mammals, including HIF-1α, -2α, and -3α. Specifically, HIF-1α is the most thoroughly studied isoform and is commonly expressed in human cells; HIF-2α is only expressed in specific tissues and cell types such as lung, kidney, and liver; and HIF-3α is mainly expressed in heart, kidney, and lung epithelial cells.^19^ Furthermore, the HIF-1β subunits, also known as the aryl hydrocarbon receptor nuclear translocator (ARNT), are encoded by two genes, ARNT1 and ARNT2.^20^^,^^21^ Reyes et al^22^ revealed that HIF-1β is stably expressed in humans and is also a specialized chaperone of the aryl hydrocarbon receptor (AHR) (Fig. 1).

### The basic structure of HIF-1α

HIF-1 belongs to an oxygen-dependent transcriptional activator widely present in human and mammalian cells and is a heterodimer composed of an α subunit (HIF-1α) and a β subunit (HIF-1β) that contributes to the progression of tumors and mammalian development.^23^ Among them, the β-subunit is a structural subunit that is continuously expressed in the nucleus independently of oxygen concentration, as compared to the α-subunit, which is a functional subunit that responds differently to hypoxia and normoxia and is the main site of HIF-1 in tumorigenesis.^3^^,^^23^

Both HIF-1α and HIF-β are classified as members of the basic helix-loop-helix (bHLH)-PAS (PER-ARNT-SIM, PAS) family of structural domain transcription factors, which contain a bHLH domain and two PAS domains (PAS-A and PAS-B).^24^^,^^25^ Typically, the HLH and PAS domains facilitate heterodimerization between HIF-1α and HIF-β, while the basic region preceding the N-terminal end of the HLH domain is responsible for the binding of the HIF-1α/HIF-β heterodimer to the DNA motifs of the hypoxia response elements (HREs) on the promoter of HIF-1 target genes.^26^^,^^27^

In addition, HIF-1α also possesses other critical structural domains, one of which is the oxygen-dependent degradation domain (ODDD) located at the free carboxyl terminus (C-terminus) of the HIF-1α peptide chain, which functions to rapidly degrade the α-subunit under normoxia^28^; and two transactivation domains (TAD), respectively near the C-terminus (C-TAD) and the free amino terminus (N-TAD), both of which are required for HIF-1α initiation and stimulation of target genes transcription.^29^ Of these, C-TAD can modulate the trans-activation of target genes through the recruitment of CBP/p300 (a transcriptional coactivator).^30^ Interestingly, there is also an inhibitory domain (ID) between C-TAD and N-TAD that can decrease the activity of the transcriptional activation domain of HIF-1α.

**Figure 2:**
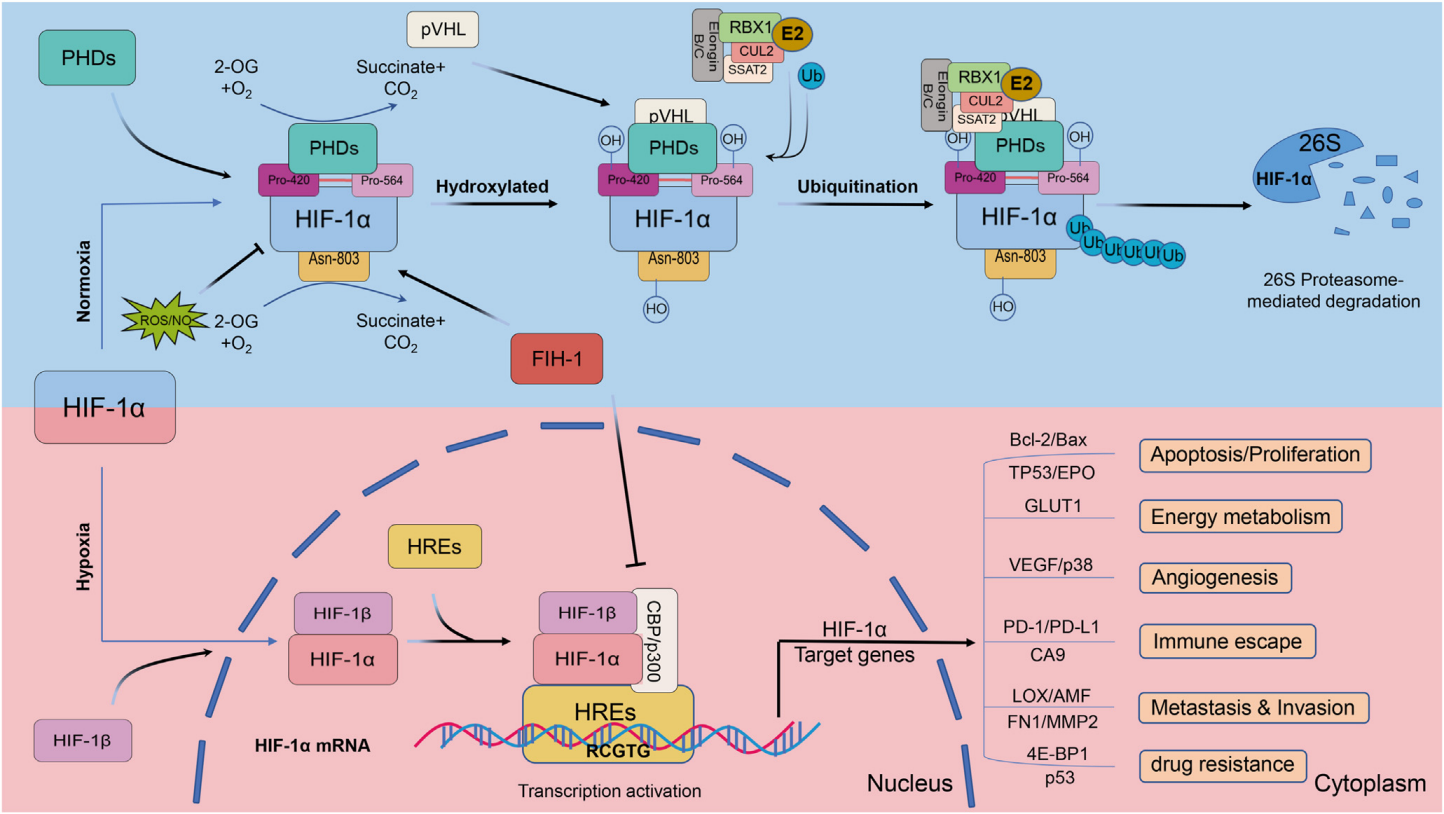
The degradation and activation of HIF-1α. Under normoxic conditions, the key residues of the conserved proline (Pro-402 and Pro-564) in the HIF-1α subunit can be hydroxylated by PHD. Subsequently, the HIF-1α subunit is ubiquitinated by the E3 ubiquitin ligase complex, which contains the Von Hippel-Lindau protein (a negative regulator of HIF-1α transcriptional activity) and is ultimately degraded by the 26S proteasome. Asparagine (Asn-803) is hydroxylated by asparagine hydroxylase, which inhibits the transactivation of HIF-1α and prevents HIF-1α from binding to CBP/p300 (transcriptional co-activator). FIH1 and ROS/NO also affect HIF-1α activity; the former blocks HIF-1α binding to CBP/p300 and inhibits HIF-1α transcriptional activation, and the latter suppresses the acetylation of HIF-1α by impeding the activation of PHD. In contrast, hydroxylation and acetylation of HIF-1α are inhibited under hypoxia. Stable HIF-1α forms a heterodimer with the HIF-1β (ARNT) subunit after translocation to the nucleus, which in turn binds to CBP/p300 and binds to hypoxia-responsive elements (HREs) on the promoter of HIF-1 target genes to constitute a transcription initiation complex, which eventually activates downstream target genes. NO, nitric oxide; ROS, reactive oxygen species.

Under normoxic conditions, it has been confirmed that HIF-1α is predominantly catabolized via the ubiquitin-proteasome pathway in normal cells and is hyperexpressed.^3^ Mechanistically, α-ketoglutarate and iron-dependent prolyl hydroxylase (PHD) catalyze the hydroxylation of proline residues (*e.g.*, Pro-402 and Pro-564) in the ODDD of HIF-1α^31^, ^32^, ^33^; concurrently, the Lys residue at position 532 is acetylated by acetyltransferase-1 (ARD-1). The residue is subsequently recognized by the Von-Hippel-Lindau (VHL) E3 ubiquitin ligase complex, leading to ubiquitination of the HIF-1α subunit and eventual degradation by the 26S proteasome, which indicates a negative regulatory effect of PHD on HIF-1α transcription.^33^^,^^34^ Moreover, the transcriptional activity of HIF, which escapes degradation, is controlled by the factor inhibiting HIF (FIH), an oxygen-dependent enzyme with asparagine hydroxylase activity towards the HIF-α subunit.^35^ The oxygen-dependent hydroxylation of asparagine residues (*e.g.*, Asn-803) disrupts the interaction between CBP/p300 and HIF-1α via FIH, thereby inhibiting its transcriptional activity.^31^^,^^35^^,^^36^ In contrast, PHD and FIH are inactive under hypoxic conditions and the asparagine and prolyl in the HIF-1α protein fail to be hydroxylated, permitting the accumulation of HIF-1α in the cytoplasm.^30^^,^^37^ Further, the stable HIF-1α subunit is importantly transferred to the nucleus to form a heterodimer with HIF-1β and binds to HREs to activate downstream target genes that are involved in various life processes such as angiogenesis, glucose metabolism, cell proliferation, and survival^31^^,^^38^, ^39^, ^40^ (Fig. 2).

### The functions of HIF-1α

To our knowledge, the expression of HIF-1α is elevated in many types of human cancer cells, and there is a definite correlation between HIF-1α and tumor prognosis.^41^ In particular, the experimental data demonstrate that HIF-1α, a key transcription factor for adaptation to hypoxia, can regulate the expression of >100 genes downstream, including four categories of target genes that are inextricably linked to protein production and tumors: vascular endothelial growth factor (VEGF), glucose transport and glycolytic enzymes (GLUT), factors involved in tumor invasion and metastasis, and proteins related to tumor proliferation and apoptosis.^42^ Notably, in the process of disease development, these genes, when affected, can modulate the relative homeostasis of the internal environment in tissues and cells under hypoxic conditions. For example, they not only modulate classical biological behaviors that include stem cell maintenance, cell survival, apoptosis, erythropoiesis, and angiogenesis, but also influence the microenvironment and metabolic state of tumors, the energy metabolism of nucleotides, amino acids, and glucose, and participate in immune evasion. Additionally, HIF-1α drastically regulates genes related to drug resistance and other altered responses and therefore acts as a key player in tumorigenesis.

### Malignant biological behaviors of HIF-1α in tumorigenesis under hypoxia

**Figure 3:**
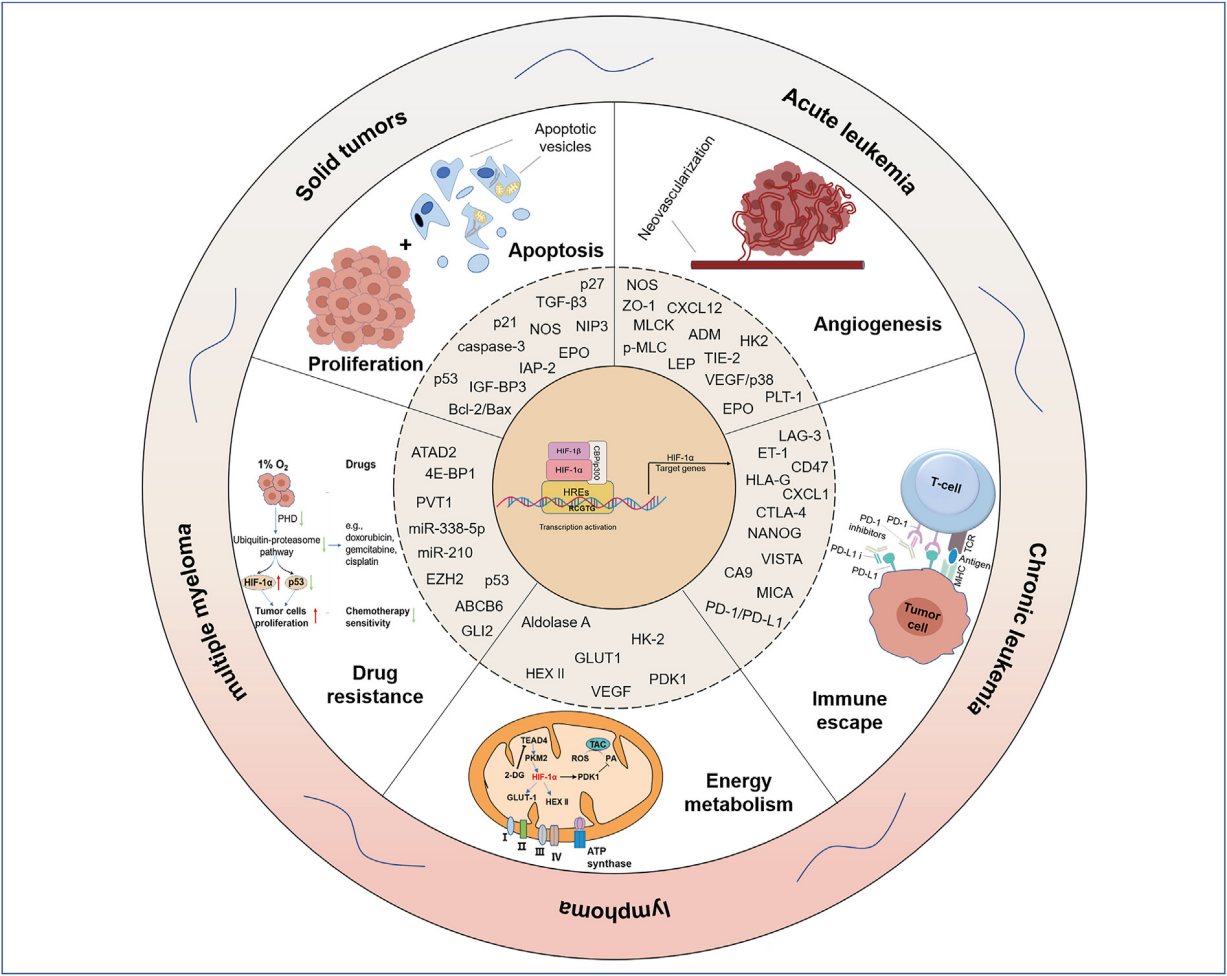
Malignant biological behaviors of HIF-1α in tumorigenesis under hypoxia. The tumor microenvironment (TME) is a complex environment in which tumor cells live. The TME plays a crucial role in tumorigenesis, growth, and metastasis. The rapid proliferation of malignant tumor cells contributes to increased consumption of oxygen, while metastasis leads to reduced oxygen delivery, thus creating a hypoxic microenvironment (HME) for tumor formation. In a hypoxic environment, the accumulation of HIF-1α can regulate the expression of downstream genes through a variety of mechanisms, promoting tumor cell proliferation, angiogenesis, energy metabolism, epithelial–mesenchymal transition (EMT), and immune escape, *etc.*, thereby making tumor cells more tolerant to HME and acquiring the greater capacity for proliferation, metastasis, and invasion.

The tumor microenvironment (TME) is the complex environment in which tumor cells survive. It contains not only the structure, function, and metabolism of the tumor tissue but also the intrinsic environment (nuclear and cytoplasmic) of the tumor cells themselves. Essentially, the TME is composed of tumor cells, stromal cells, endothelial cells, and immune cells.^43^ In the process of tumor occurrence, as well as growth and metastasis, TME plays a crucial role. The rapid proliferation of malignant tumor cells contributes to increased consumption of oxygen, and metastasis in turn results in reduced oxygen delivery, thus creating an HME for tumor formation. In a hypoxic environment, the accumulation of HIF-1α can regulate the expression of downstream genes through multiple mechanisms, facilitating tumor cell proliferation, angiogenesis, energy metabolism, epithelial–mesenchymal transition (EMT), immune escape, *etc.*, thereby making tumor cells more tolerant of the HME and acquiring greater capabilities for proliferation, metastasis, and invasion^42^^,^^44^ (Fig. 3).

### HIF-1α and the proliferation and apoptosis of tumor cells

In the HME, mounting evidence has indicated that HIF-1α can modulate downstream target genes to promote the proliferation of tumors as well as suppress the apoptosis of tumor cells via interacting with cofactors.^45^^,^^46^ JAG1 (JAG1), a specific ligand for the Notch signaling pathway in mammals, acts in interaction with Notch receptors to activate the Notch pathway, provoking the shedding of the Notch intracellular structural domain (NICD), and binding to cofactors to stimulate the expression of numerous target genes involved in diverse life processes.^47^^,^^48^ More importantly, it has been reported that hypoxia-activated HIF-1α participates in tumor metabolism and angiogenesis, and boosts the proliferation, invasion, and metastasis of tumors by activating the Notch pathway, a key regulatory signaling mechanism that governs the fate of tumor cells and EMT.^49^^,^^50^ Conversely, inhibition of the Notch pathway contributes to further apoptosis of tumor cells. As is widely known, the *TP53* gene is primarily responsible for encoding the corresponding tumor suppressor protein, p53, and is the most commonly mutated gene in all human cancers. Indeed, previous reports have declared that wt p53 (wild-type p53, wt p53) negatively regulates the stability of HIF-1α. Thus, Amelio et al^51^ showed that the main role of wt p53 is to modulate the cell cycle and accelerate cell death and that the physical binding of HIF-1α to wt p53 facilitates wt p53-mediated hypoxic cell death. In addition, cancer cells are often already exposed to decreased oxygen tension at the stage when mutations in *TP53* occur.^52^^,^^53^ Mutant-type p53 (mt p53) can accelerate cell growth via interacting with HIF-1α and is ultimately engaged in the progression of tumors.^51^ Furthermore, HIF-1α also inhibits the apoptosis of tumor cells by down-regulating the levels of the anti-apoptotic proteins Bcl2 (*e.g.*, IL-6/STAT3/Bcl2 pathway),^54^^,^^55^ an inhibitor of apoptosis protein-2 (IAP-2)^55^ and lowering the levels of cleaved caspase-3 and the pro-apoptotic proteins Bcl2 associated X protein (BAX).^56^

### HIF-1α and energy metabolism in malignant tumors

Specifically, many critical enzymes of the glycolytic pathway are targets of HIF-1α, which is thought to mediate the activation of glycolysis in cancer cells. Under conditions of severe hypoxia, HIF-1α prevents the entry of pyruvate into the tricarboxylic acid cycle and elevated mitochondrial reactive oxygen species (ROS) by activating the PDK1 protein, avoiding a massive increase in ROS to cause apoptosis.^57^ Here, Semba et al^57^ consider that the metabolic changes are related to the adaptation of tumor cells to a hypoxic environment. In another recent study, the researchers found that up-regulation of HIF-1α in tumor cells is resistant to apoptosis and proliferation hindrance induced by hypoxia and hypoglycemia, while inhibition of HIF-1α expression makes cancer cells more sensitive to apoptosis and proliferation hindrance induced by hypoxia and hypoglycemia.^58^ Mechanistically, reduced expression of Glut-1 and aldolase A mRNA and sugar uptake-related proteins in tumor tissues would result in decreased tumorigenicity of *in vivo* tumor cells.^58^ Thus, these results suggest that the effect of HIF-1α on cancer cells relies on alterations in glycolytic metabolism and is a potentially effective way to cure tumors. Pyruvate kinase isozymes M2 (PKM2) is a key regulatory enzyme in glycolysis and was predicted to be a target of transcription factor TEA domain 4 (TEAD4) by Hu et al^59^ using bioinformatics analysis. In *in vitro* experiments on tumor cells, they have confirmed, using a luciferase reporter gene assay, that TEAD4 boosts the transcription and expression of PKM2, which further elevates the activity of HIF-1α and therefore increases the expression of the HIF-1α-targeted glycolytic genes glucose transporter-1 and hexokinase II; conversely, the supplementation of 2-deoxy-d-glucose (2-DG) blocked the positively regulated glycolysis by TEAD4 and PKM2.^59^ Furthermore, peroxisome proliferator-activated receptors (PPAR) are ligand-activated nuclear receptors of the steroid/thyroid hormone receptor superfamily. There are three isoforms, α, β/δ, and γ, which are closely associated with lipid storage and mobilization, glucose metabolism, morphogenesis, and inflammatory responses.^60^ Among them, the expression of PPAR-γ was known to be modulated by HIF-1α.^61^ Simvastatin, a cholesterol-lowering drug, can suppress the HIF-1α/PPAR-γ/PKM2 axis via inhibiting PKM2-mediated glycolysis, leading to reduced proliferation and increased apoptosis in HCC cells and re-sensitizing HCC cells to sorafenib.^62^ Collectively, these data provide a theoretical basis for implicating HIF-1α as a poor prognosticator.

### HIF-1α and tumor angiogenesis

Malignant tumorigenesis is typically accompanied by a marked hypoxia in the tumor environment, which may be related to insufficient tissue oxygenation due to the excessive growth of tumors and relatively low angiogenesis. However, to the extent that tumor tissue can continue to grow in this hypoxic environment, HIF-1α performs an essential role in the process. Vascular endothelial growth factor (VEGF) acts as a key mediator of angiogenesis, mainly promoting the proliferation, survival, and migration of endothelial cells, and increasing vascular permeability.^63^ Some previous studies revealed that HIF-1α has been identified as a major regulator of VEGF expression. In a hypoxic environment, the HIF-1α-p300/CBP complex binds to the HRE sequence in the 5′ promoter region of the *VEGF* gene, thus triggering the expression of *VEGF* and neovascularization.^64^ Currently, it is widely acknowledged that PIM kinases are oncogenic serine/threonine kinases.^65^ Increased expression of PIM1 *in vivo* can trigger the expression of HIF-1α target genes (*VEGF* and *HK2*) by directly phosphorylating HIF-1α at threonine 455, thereby inducing angiogenesis; however, *VEGF* is notably deficient in HIF-1α-knockdown tumor tissues.^66^ Prolactin II (SCG2) is markedly under-expressed in malignant tumor tissues and is correlated with shorter disease-free survival and overall survival in cancer patients.^67^, ^68^, ^69^ In another animal study, SCG2 suppressed the expression of *VEGF* via interacting with VHL tumor inhibitors in cancer cells, fostering the degradation of HIF-1α; in contrast, the accumulation of HIF-1α could effectively elevate the SCG2-mediated expression of *VEGF* in cancer cells.^67^ Hence, it has been revealed that ectopic expression of SCG2 apparently impeded tumor growth by disrupting angiogenesis.

### HIF-1α and tumor immune escape

In the TME, HIF-1α mainly promotes tumor immune escape by regulating a variety of immune cells (*e.g.*, T lymphocytes, macrophages, myeloid-derived suppressor cells (MDSCs)).^70^, ^71^, ^72^ Dysregulation of the immune microenvironment and treatment resistance are the dominant causes of cancer. Concretely, the programmed cell death protein 1 (PD-1)/programmed cell death 1 ligand 1 (PD-L1) axis is an integral component of the immunosuppression in the organism.^73^ In 2021, Deng et al^73^ showed that HIF-1α can result in tumor immune escape by mediating the up-regulation of PD-L1, which in turn leads to disease progression. Furthermore, suppression of the PD-1/PD-L1 axis may alleviate chemoresistance in tumor patients due to the guaranteed survival of CD4^+^ T cells and CD8^+^ T cells.^73^ Meanwhile, PD-1 has been reported for the first time as a novel downstream molecule of HIF-1α, apart from PD-L1.^74^ However, the efficacy of anti-PD-1 therapies for tumors with high HIF-1 levels remains to be explored. Another recent study found that HIF-1α prevents tumor cells from being subjected to immune surveillance with enhanced levels of carbonic anhydrase IX (CA9) expression.^75^ More importantly, it is apparent that fenofibrate, a PPAR alpha (PPAR-α)-specific agonist, further represses the expression of hypoxia-induced HIF-1α and CA9 in tumors by activating the AMP-activated protein kinase (AMPK) pathway and SIRT1 in tumor cells using HO-1.^75^ Overall, the research into drugs targeting HIF-1α-related immune mechanisms could be of assistance in anti-cancer therapy.

### HIF-1α and tumor drug resistance

Under hypoxic conditions, the sensitivity of tumor cells to chemotherapeutic drugs is greatly impaired. HIF-1α and p53 are jointly engaged in anti-cancer drug resistance. The cytotoxicity of anticancer drugs (*e.g.*, doxorubicin, gemcitabine, and cisplatin) at 1% oxygen was even lower than that at 5% oxygen, mainly owing to the fact that these drugs increased the expression of 4E-BP1-dependent HIF-1α protein, but did not alter the level of p53.^76^ However, when oxygen concentrations were raised, the drugs not only inhibited HIF-1α expression but also enhanced p53 levels, ultimately resulting in massive death of tumor cells.^76^ Recently, accumulating evidence reveals that microRNAs (miRNAs) play an influential role in the acquired drug resistance of tumor cells.^54^ In *in vitro* cellular assays, overexpression of HIF-1α reduced the expression of miR-338-5p under both normoxic and hypoxic conditions. Subsequently, the deficiency of miR-338-5p in hypoxic cancer cells moderated the decrease in IL-6 expression and reactivated the anti-apoptotic STAT3/Bcl2 signaling pathway, thus impeding tumor cell death and improving the drug resistance of tumor cells.^54^ Taken together, the Yin group here has identified a hypoxia-triggered HIF-1α/miR-338-5p/IL-6 feedback loop, offering a direction for future research in cancer therapy.

### The role of HIF-1α in solid tumors

**Figure 4:**
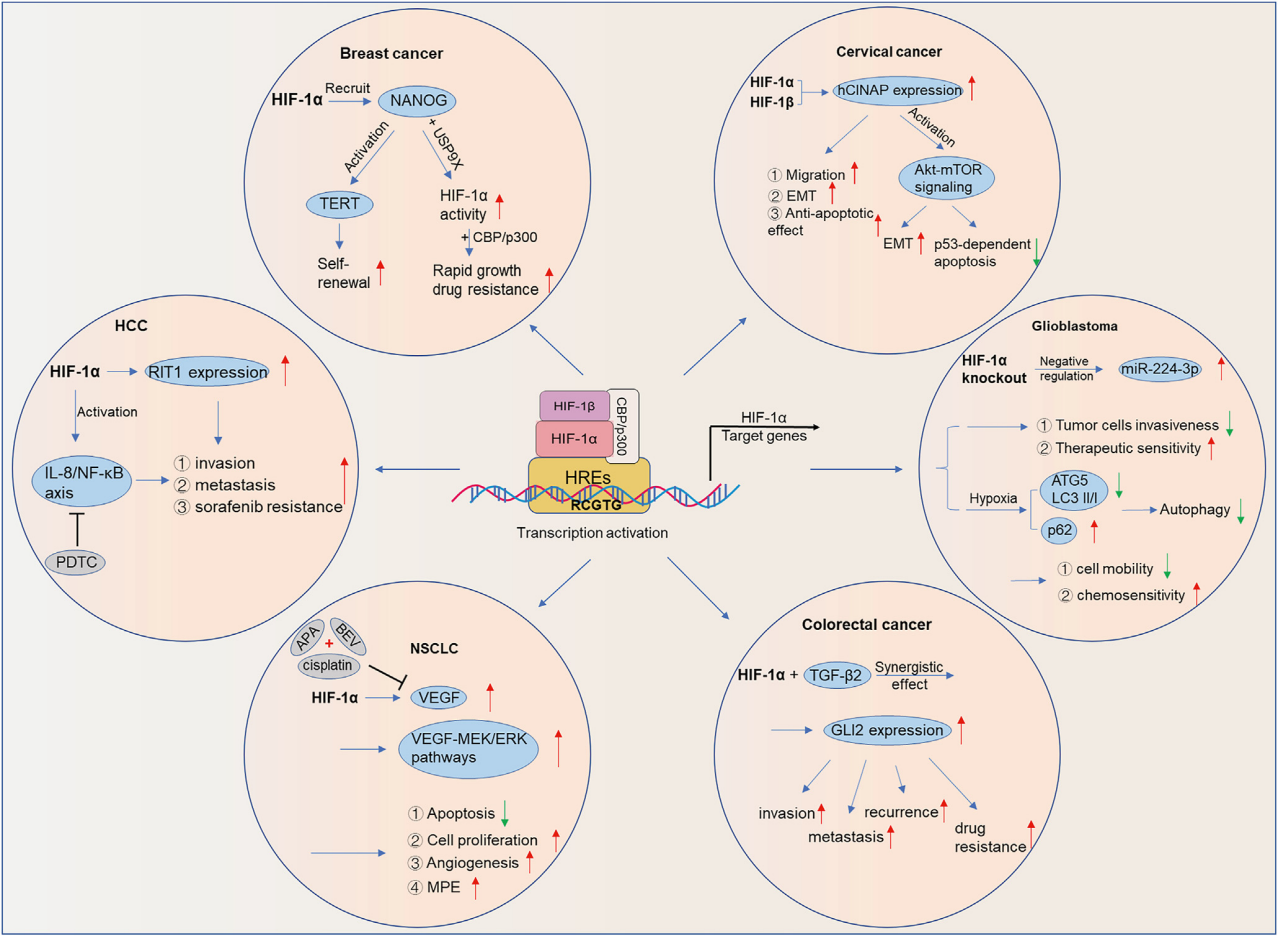
The role of HIF-1α in solid tumors. HCC, hepatocellular carcinoma; NSCLC, non-small-cell lung cancer; EMT, epithelial-to-mesenchymal transition; hCINAP, human coilin-interacting nuclear ATPase protein; MPE, malignant pleural effusion; BEV, bevacizumab; VEGF, vascular endothelial growth factor; PDTC, pyrrolidine dithiocarbamate; APA, apatinib.

In the hypoxic environment triggered by solid tumors, HIF-1α is stably present and activates the transcription of tumor invasion-related genes, thus fostering tumor progression and chemotherapy resistance.^77^ In brief, tissue hypoxia and certain molecular alterations (*e.g.*, mutations in oncogenes such as *ERBB2* and suppressors such as *VHL* and *PTEN*) contribute to the overexpression of HIF-1α in tumor cells, which in turn leads to tumor metastasis, treatment failure, and high mortality^37^^,^^78^, ^79^, ^80^, ^81^ (Fig. 4).

It has been reported that HIF-1α is highly expressed in tumors such as breast, liver, bladder, cervical, colorectal, endometrial, lung, *etc*.^77^ NANOG is a protein required for the self-renewal of breast cancer stem cells (BCSCs), and high expression of NANOG in tumor tissues of breast cancer patients is correlated with poor prognosis.^82^ Mechanistically, HIF-1α maintains telomere length by recruiting NANOG to activate transcription of the TERT gene (encoding telomerase reverse transcription), ensuring self-renewal in stem cells. In addition, NANOG also binds to the deubiquitinase USP9X to regulate HIF-1α activity and consequently stimulate the rapid growth of tumors and drug resistance through the interaction of HIF-1α with CBP/p300.^82^ In HCC, HIF-1α could promote the growth, invasion, metastasis, and development of drug resistance to sorafenib treatment in HCC cells by up-regulating the expression of the target gene *RIT1*.^83^ Similarly, Feng et al^84^ found that HIF-1α was significantly overexpressed in HCC cells and facilitated the migration and invasion of HCC cells by modulating IL-8 via the NF-κB (nuclear factor-kappa B) pathway. Pyrrolidine dithiocarbamate (PDTC), an inhibitor of NF-κB, could block the HIF-1α/IL-8/NF-κB axis and thus inhibit the proliferation of HCC cells. In cervical cancer cells, HIF-1α and HIF-1β facilitate the migration capacity, EMT, and anti-apoptotic effects of cancer cells in response to hypoxia via directly up-regulating the expression of its target gene *hCINAP*.^85^

Owing to the survival of cancer stem cells (CSC), colorectal cancer patients generally suffer recurrence after chemotherapy. Recent studies have confirmed that high expression levels of HIF-1α/TGF-β2/GLI2 are closely associated with relapse after chemotherapy in patients.^86^ Inhibition of HIF-1α could effectively reverse chemoresistance induced by the TME by blocking TGF-β2/GLI2 signaling, indicating a potential biomarker and therapeutic target.^86^^,^^87^ In the glioma LN229 cell line, knockdown of HIF-1α inhibited cell invasiveness and increased therapeutic sensitivity by negatively modulating the time-dependent expression of miR-224-3p in hypoxia.^88^ Moreover, further studies revealed that hypoxia can increase the relative expression of ATG5 (autophagy-associated gene 5), which is a target of miR-224-3p, suggesting that it induces autophagy in tumor cells, eventually causing them to be less sensitive to chemotherapeutic drugs.^88^ Using a mouse xenograft model of non-small cell lung cancer (NSCLC), Xiang et al^89^ observed that a triple–drug combination (apatinib + bevacizumab (BEV) + cisplatin) suppressed angiogenesis and the formation of malignant pleural effusion (MPE) in NSCLC by down-regulating HIF-1α, VEGF, VEGFR-2, MEK1, and MMP-2 molecular signaling pathway proteins, and facilitated apoptosis of tumor cells *in vivo*, ultimately greatly prolonging the overall survival of tumor-bearing mice. Apart from the above, HIF-1α also participated in the proliferation, migration, invasion, and angiogenesis of tumor cells in cholangiocarcinoma.^90^

### The role of HIF-1α in leukemia

Indeed, studies have confirmed that the oxygen concentration in the bone marrow (BM) is extremely rare, down to 0.6%, and that the hypoxic environment is protective of leukemic stem cells and maintains their ability to self-renew.^91^ As is well known, the testis and the central nervous system (CNS) are the most vulnerable sites for extramedullary relapse of acute lymphoblastic leukemia (ALL), and both have a common feature with the BM — a hypoxic environment. Importantly, hypoxia-activated HIF-1α can affect the prognosis of patients with leukemia^91^ (Table 1).

**Table 1:** The functional roles of HIF-1α in diverse types of hematological malignancies.

Cancer type	Patients or cell lines	Role of HIF-1α in cancer	Functions	Molecular mechanisms	Reference
ALL	ALL cells/mice	Oncogene	Deferoxamine (DFO) inhibited the proliferation and growth of tumor cells and induced apoptosis in ALL cells by inactivating ROS/HIF-1α signaling.	DFO/ROS/HIF-1α	^92^
	BM samples of ALL patients/leukemia cell lines	Oncogene	Chemical inhibition of HIF-1α induced down-regulation of YY1, sensitizing cells to chemotherapeutic agents.	HIF-1α/YY1	^93^
	Human T-ALL cell samples/mice	Oncogene	HIF-1α KD restored the activity of mTOR at low oxygen concentrations, thereby regaining chemosensitivity in T-ALL cells.	HIF-1α/mTOR	^94^
CLL	lines/mice	Oncogene	SC could induce up-regulation of HIF-1α in CLL cells, ultimately promoting the survival of CLL cells.	HIF-α/CXCL12/CXCR4	^95^
	PB samples of CLL/cell lines/mice	Oncogene	In the *TP53*-disrupted (*TP53*^dis^) subset, the accumulation of HIF-1α led to reduced apoptosis and drug resistance in CLL cells when hypoxic.	pVHL/HIF-1α	^96^
	CLL patients/a murine model	Oncogene	The relevant drugs inhibited the growth of CLL cells by suppressing HIF-1α and interfering with intracellular redox homeostasis.	HIF-1α/oxidative stress	^97^
	Primary CLL cells/CLL cell lines/mice	Oncogene	EZN-2208, confirmed to inhibit HIF-1α, could increase the response to fludarabine by promoting apoptosis in CLL cells.	EZN-2208/HIF-1α	^98^
AML	AML patients/AML cells/mice	Oncogene	PARP14 induced the growth of AML cells and glycolysis via activating NF-κB and facilitating the expression of HIF-1α.	PARP14/NF-κB/HIF-1α	^99^
	Cell lines	Oncogene	Simvastatin inhibited the proliferation, migration, and invasion, and promoted apoptosis of AML cells by modulating the miR-19a-3p/HIF-1α axis.	Simvastatin/miR-19a-3p/HIF-1α	^45^
	THP-1 and HL-60 cells	Oncogene	Under hypoxia, CXCL2 promoted the proliferation and migration of AML cells by enhancing the activity of HIF-1α and up-regulating PIM2 expression.	CXCL2/HIF-1α/PIM2, mTOR	^100^
	AML cells	Oncogene	Inhibition of HIF-1α could down-regulate the expression of YAP in AML cells, thereby re-sensitizing AML cells to ADR in hypoxia.	HIF-1α/YAP	^101^
	AML patients/AML cell lines/mice	Oncogene	Echinomycin could effectively treat patients with *TP53*-mutated AML via inhibiting HIF-1α.	Echinomycin/HIF-1α	^102^
	Healthy donor mesenchymal stromal cells/patient samples/AML cell lines	Oncogene	Abrogation of STC1 (stanniocalcin 1) or HIF-1α attenuated the inhibition of HSPC differentiation and proliferation by AML.	HIF-1α/STC1	^103^
CML	TKIs sensitive and resistant CML cells	Oncogene	HIF-1α could facilitate the expression of BCR-ABL1 and Met to rescue CML cells from death.	HIF-1α/BCR-ABL1, Met	^104^
	K-562 cells	Oncogene	2-methoxyestradiol (2-ME2) induced apoptosis in CML cells by suppressing the expression of HIF-1α and down-regulating C-Myc or Bcl-xl and Bcl-2 genes.	2-ME2/HIF-1α/C-Myc, Bcl-xl or Bcl-2	^105^
	CML patients/CML cell lines/mice	Oncogene	Tri-CAP (trident cold atmospheric plasma) could disrupt cancer survival pathways such as proliferative AKT/mTOR/HIF-1α signaling, thereby inducing apoptosis of tumor cells.	Tri-CAP/AKT/mTOR/HIF-1α	^106^
lymphoma	T-cell lymphoma cells	Oncogene	Sildenafil suppressed the expression of HIF-1α and decreased glucose metabolism, thus enhancing the killing ability of cisplatin on tumor cells.	HIF-1α/glycolysis regulatory molecules/ROS	^107^
	Mice	Oncogene	In *in vivo* experiments, MJ promoted T-cell lymphoma cell death by docking with prominent binding sites for HIF-1α, HK2, and Hsp70.	MJ/HIF-1α	^108^
	Tumor cell line Hut78/mice	Oncogene	Inhibition of HIF-1α blocked the glycolysis and IL-17 pathways induced by CHOP chemotherapy.	HIF-1α/glycolysis/IL-17	^109^
MM	MM cell lines	Oncogene	Macitentan down-regulated HIF-1α and the transcription and release of downstream pro-angiogenic cytokines, curbing the growth of MM cells.	ET-1/HIF-1α/pro-angiogenic cytokines	^110^
	MM patients/mice	Oncogene	Low expression of miR-411-3p could promote malignant proliferation and tumor stem cell-like properties of MM by activating HIF-1α.	lncRNA ANRIL/miR-411-3p/HIF-1α	^111^

Abbreviations: DFO: Deferoxamine; mTOR: rapamycin; SC: stromal cell; BM: bone marrow; PB: peripheral blood; pVHL: von Hippel-Lindau tumor suppressor protein; YAP: yes-associated protein; ADR: adriamycin; 2-ME2: 2-Methoxyestradiol; MSCs: mesenchymal stromal cells; STC1: stanniocalcin 1; Tri-CAP: trident cold atmospheric plasma; PIM2: proviral integration moloney 2; ROS: reactive oxygen species; HK2: hexokinase 2; MJ: methyl jasmonate.

### Acute lymphoblastic leukemia (ALL)

T-cell acute lymphoblastic leukemia (T-ALL) is a highly aggressive hematological malignancy. Based on the analysis of relevant experiments with T-ALL cells *in vivo* and *in vitro*, Fahy et al^94^ revealed that hypoxia strongly suppresses the growth of CD45/CD7 T-ALL cells, which renders them insensitive to anti-leukemic drugs but retains their proliferative potential after the end of treatment. Further studies found that knockdown (KD) of HIF-1α counteracts the effects noted in hypoxic T-ALL and regains their chemosensitivity. Furthermore, activation of mammalian rapamycin (mTOR) was attenuated and drug resistance increased in hypoxic T-ALL cells; meanwhile, HIF-1α KD also restored mTOR activity at hypoxic concentrations and suppression of mTOR in HIF-1α KD T-ALL impaired the chemotherapeutic effect in leukemic cells.^94^ Therefore, activation of the HIF-1α/mTORC1 axis under hypoxia can cause growth inhibition of leukemic cells, resulting in the development of drug resistance. Similarly, in another T-ALL study, Notch1 has been reported to function as an oncogenic player in the disease process.^112^ Using small interfering RNA (siRNA) transfection, silencing HIF-1α suppressed Notch1 signaling under hypoxic conditions, as evidenced by reduced expression of its downstream target gene *Hes1*, which inhibited cell proliferation, invasion, and chemoresistance; in contrast, silencing Notch1 did not affect the expression of HIF-1α.^112^ Yin-Yang transcription factor 1 (YY1) is elevated in different cancers, including leukemia, and is negatively correlated with prognosis.^113^ Importantly, the B-ALL cell line RS4;11 showed that both HIF-1α and YY1 proteins are co-expressed in response to hypoxia. Conversely, inhibition of HIF-1α expression levels triggered the down-regulation of YY1, resulting in increased sensitivity of tumor cells to chemotherapeutic agents.^93^ In peripheral blood (PB) and BM samples from ALL patients, the researchers also observed a positive regulation of YY1 expression with HIF-1α.^93^ Overall, these results provide the first evidence that YY1 can be transcriptionally modulated by HIF-1α to exert an oncogenic effect.

### Chronic lymphocytic leukemia (CLL)

The HME of leukemia up-regulates HIF-1α and stimulates CLL cell survival and proliferation. In a previous study, Griggio et al^96^ found that leukemia cells of CLL patient origin with *TP53* (tumor suppressor gene) deficiency have higher expression and transcriptional activity of HIF-1α, which contributes to tumor progression, and therefore this group of patients is exposed to a worse prognosis. Consistently, they also observed that CLL cell lines cultured under hypoxia or co-cultured with stromal cells can further heighten the expression of HIF-1α. BAY87-2243 is a selective HIF-1α inhibitor that can effectively inhibit HIF-1α protein levels in tumor cells.^114^ Evidence from both *in vitro* and *in vivo* experiments indicates that BAY87-2243 has anti-tumor effects and enhances the efficacy of fludarabine and ibrutinib in CLL, independent of functional *TP53*.^114^ Hence, Seiffert concluded that CLL cells whose *TP53* was destroyed by HIF-1α inhibition are sensitive to fludarabine therapy. HIF-1α is also a novel modulator of CLL cell-TME interactions. In CLL cells, HIF-1α can regulate the expression of chemokine receptors and cell adhesion molecules, which in turn govern the interaction of cancer cells with the BM and spleen microenvironment.^115^ In a mouse model of CLL, the researchers also demonstrated that inactivation of HIF-1α attenuates the chemotaxis and adhesion of leukemic cells to the stroma, reduces their colonization of the BM and spleen, and potentially prolongs the survival of mice.^115^ In another study, hypoxia-mediated overexpression of HIF-1α increased the production and signaling of adenosine as well as protecting against drug-driven apoptosis in CLL cells, thus ultimately facilitating tumor progression.^116^ However, blockade of the A2A adenosine receptor could abrogate the effects of HIF-1α in tumor cells, allowing leukemic cells to regain sensitivity to therapeutic agents.^116^ Explicitly, targeting HIF-1α or its downstream regulatory pathways (*e.g.*, CXCL12/CXCR4 axis) at the tumor and stromal cell levels may be a viable strategy to surmount HME-mediated protection of CLL cells.^95^

### Acute myeloid leukemia (AML)

In AML, leukemic stem cells (LSCs) can favor the constant renewal of tumor cells, leading to suffering from chemoresistance and a high relapse rate in treated patients, with a poor prognosis for the majority of patients.^101^^,^^117^ In the current study, Zhu et al^101^ concluded that overexpression of HIF-1α decreased the sensitivity of AML cells to adriamycin (ADR) under hypoxia and assisted in maintaining ADR resistance. However, the HIF-1α inhibitor CdCl2 remarkably inhibited the proliferation of AML cells by restoring their sensitivity to ADR in the presence of hypoxia. Further studies indicated that induction of HIF-1α significantly elevates the expression of yes-associated protein (YAP) in AML cells, and more markedly in drug-resistant cells.^101^ Targeting the HIF-1α/YAP regulatory loop could effectively improve the efficacy of ADR-based AML chemotherapy. In the BM microenvironment, HIF-1α can up-regulate the expression of the AML-derived macrophage migration inhibitory factor MIF, thereby facilitating the proliferation and survival of AML cells.^118^ Functionally, inhibition of MIF or HIF-1α *in vivo* significantly attenuated the tumor burden in the BM and prolonged the lifespan of AML mouse models.^118^

Acute promyelocytic leukemia (APL) belongs to a class of AML characterized by the oncogenic fusion protein PML-RARα generated by the t (15;17) chromosome translocation. In APL, HIF-1α can act in concert with PML-RARα to function as a transcriptional co-activator.^119^ In *in vitro* and *in vivo* studies, it has been identified that PML-RARα boosts pro-leukemic functions driven by HIF-1α, including cell migration, BM neo-angiogenesis, and self-renewal of tumor cells. In contrast, deletion of HIF-1α in APL cells dramatically impaired the migration, chemotaxis, and invasion of leukemic cells.^119^ In AML, *TP53* mutation predicts a poor prognosis for the disease. In another study, Wang et al^102^ revealed that the HIF-1α inhibitor echinomycin significantly suppressed the proliferation of *TP53*-mutated AML stem cells. Furthermore, they observed that echinomycin monotherapy proved to be more effective in killing AML cells than conventional combination chemotherapy (cytarabine plus daunorubicin) via the establishment of a mouse model of *TP53*-mutated AML.^102^ In addition, a clinical study demonstrated that HIF-1α is highly expressed in the BM of AML patients; however, when treated with evofosfamide (TH-302), the expression level of HIF-1α was notably decreased.^120^ As discussed above, HIF-1α plays an essential oncogenic role in the development of AML. Surprisingly, in some of the examined models, the deficiency of HIF-1α causes a more rapid progression of AML.^121^^,^^122^ For instance, repression of HIF-1α in an *MLL-AF9*-driven mouse model of AML did not ameliorate the efficacy of chemotherapy and instead may contribute to disease progression.^121^ Hence, these findings imply that the role of HIF-1α needs to be prudently considered in practical applications depending on the specific circumstances.

### Chronic myeloid leukemia (CML)

The oncogenic fusion gene *BCR-ABL* is one of the crucial molecular biological features that are responsible for the pathogenesis and therapeutic resistance of CML patients and activates diverse signaling pathways related to the proliferation and survival of tumor cells, such as the JAK/STAT pathway.^123^ Currently, tyrosine kinase inhibitors (TKIs) [*e.g.*, imatinib (IM)] have been proven to be effective in the treatment of CML.^123^^,^^124^ In 2010, Zhao et al^125^ observed that in the absence of BCR-ABL drug resistance mutations, HIF-1α can stimulate higher levels of BCR-ABL expression in CML cell lines, leading to leukemic cells exhibiting enhanced resistance to IM. Mechanistically, HIF-1α up-regulates the expression level of BCR-ABL by accelerating the rate of glycolysis and therefore functions as an essential anti-drug factor.^125^ Significantly, oxythiamine is capable of inhibiting HIF-1α-induced glycolysis, which in turn enhances the sensitivity of tumor cells to drugs.^125^ Similarly, in the CML cell line K562, HIF-1α could strengthen the invasiveness of cancer cells by elevating the level of glycolysis in response to the knockdown of fumarate hydratase (FH) expression, while also entailing a reduced ability to repair DNA after damage.^126^ Under this dual effect, the function of FH is disrupted and contributes to disease progression in CML.^126^ Additionally, Siah2 is highly expressed in K562-IM-resistant cells (K562-R cells).^127^ Vitamin K3 (a Siah2 inhibitor) improves the chemosensitivity of CML cells in the HME via targeted inhibition of the Siah2-PHD3-HIF-1α-VEGF axis.^127^ On the other hand, curcumin directly represses the activity of HIF-1α and interferes with the metabolic mechanisms of tumor cells.^124^ The combination of both with TKIs may potentiate the efficacy of IM. In BM specimens from CML patients, the expression level of HIF-1α mRNA was markedly higher than that of healthy controls.^128^ Consistently, in *in vitro* experiments, HIF-1α deficiency down-regulated mRNA and protein expression in p21 and p53 in K562 cells, culminating in the suppression of CML cell proliferation.^128^

### The role of HIF-1α in lymphoma

Diffuse large B-cell lymphoma (DLBCL) is the most frequently aggressive form of non-Hodgkin's lymphoma (NHL), accounting for approximately 30% of all NHL.^129^ Previous studies have shown that HIF-1α is stably expressed in most DLBCL patients.^130^ One study examined the expression levels of HIF-1α protein in 153 patients with DLBCL treated sequentially with cyclophosphamide, doxorubicin, vincristine, and prednisone (CHOP) or rituximab-CHOP (R-CHOP), and the results indicated that HIF-1α is correlated with the outcomes of DLBCL patients.^130^ In the R-CHOP group, progression-free survival (PFS) and overall survival (OS) were dramatically superior in patients with low HIF-1α expression than in those with high HIF-1α expression, while there was no survival difference in CHOP-treated patients.^130^ This finding highlights that HIF-1α is a critical prognostic factor in evaluating the likelihood of survival in DLBCL patients treated with R-CHOP. Moreover, the expression of glyceraldehyde-3-phosphate dehydrogenase (GAPDH) is elevated in NHL cell lines.^131^ Indeed, GAPDH activates NF-κB signaling by interacting with tumor necrosis factor receptor-associated factor-2 (TRAF2) and further potentiates the transcription and activity of HIF-1α factor, leading to increased aggressiveness and angiogenesis of tumors. In addition, the study has reported that elevated levels of GAPDH mRNA expression in biopsied tissues from DLBCL patients similarly can induce high levels of HIF-1α, VEGF-A, *etc*.^131^

Apart from the above, accumulating evidence has shown that HIF-1α remains dominant in the pathogenesis of other types of lymphoma. In Hodgkin's lymphoma (HL) cells, HIF-1α is predominantly expressed in the hypoxic side population (SP).^132^ Under normoxia, the production of hydrogen peroxide in HL cell lines induces cell differentiation into the major population (MP) (with giant Hodgkin and Reed-Sternberg like cells), whereas the HIF-1α stabilizer, CoCl2, counteracts the effects of hydrogen peroxide. Further studies found that heme oxygenase-1 (HO-1), triggered by HIF-1α, scavenges intracellular ROS from SP and inhibits the differentiation of tumor cells, leaving patients with a poor prognostic outcome.^132^ In *in vivo* experiments of mice, methyl jasmonate (MJ) promotes the death of T-cell lymphoma cells by binding to the action sites of HIF-1α, hexokinase 2, and Hsp70. Furthermore, MJ can relieve the chemoresistance role of HIF-1α on lymphoma cells in *in vitro* experiments.^108^ Primary effusion lymphoma (PEL) is an aggressive B-cell lymphoma due to the infection of Kaposi's sarcoma-associated herpesvirus (KSHV).^133^ Specifically, KSHV infection increases the activity of HIF-1α, which in turn activates the KSHV-encoded oncogene and maintains the optimal growth and metabolic state of PEL.^133^ Using the virus-negative Burkitt’s lymphoma cell line BJAB, which does not express HIF-1α under normoxic conditions, as a control, HIF-1α in the PEL cell lines BCBL-1 and BC-3 could remarkably affect the growth metabolism, viral replication, and expression of viral-encoded oncogenes in PEL cells.^133^ Thus, these results reflect that HIF-1α exerts an influential role in the PEL.

### The role of HIF-1α in multiple myeloma (MM)

In MM cells, the endothelin-1 (ET-1) receptor, by binding to autocrine ET-1, can act to prolong the survival of tumor cells.^110^ In essence, ET-1-mediated expression of HIF-1α prominently affects the release of pro-angiogenic cytokines (*e.g.*, VEGF-A, IL-8, and ET-1 itself), thereby triggering the proliferation, invasion, and extramedullary metastasis of MM cells (*e.g.*, RPMI-8226). Upon treatment with macitentan, a dual ET-1 receptor antagonist, Russignan et al^110^ found that HIF-1α expression is down-regulated and reverses the effects of ET-1. More importantly, macitentan was equally effective in inhibiting the proliferation and microvascular density of tumor cells in animal experiments. Multidrug resistance is commonly associated with poor prognosis in MM patients. In an *in vitro* study, a single and combined treatment of MM cell lines RPMI8226/L-PAM and ARH-77/L-PAM with MEK inhibitors, PI3K inhibitors, and NF-κB inhibitors induced melphalan sensitization and markedly down-regulated the expression of HIF-1α.^134^ Further studies revealed that RPMI8226/L-PAM resistance to melphalan was correlated with the activation of ERK1/2, Akt, and NF-κB to entail the up-regulation of HIF-1α, which consequently transcriptionally activates the target gene *Survivin* and decreases the expression of Bim.^134^ In summary, these findings demonstrate that targeting HIF-1α, ERK1/2, Akt, and NF-κB has the potential to be used in the treatment of drug-resistant MM. Consistently, overexpression of tripartite motif-containing 44 (TRIM44) in MM cells is also crucial for treatment resistance in MM patients.^135^ In a MM xenograft mouse model, the deubiquitinase TRIM44 promotes the quiescence of stem cancer cells; on the other hand, TRIM44 can spur the proliferation and survival of MM cells under hypoxia via elevating the expression level of HIF-1α, which ultimately exerts a drug-resistant and oncogenic effect.^135^

### The role of HIF-1α in cancer stem cells (CSCs)

CSCs, also known as tumor-initiating cells, are a subpopulation of tumor cells that exhibit properties similar to those of normal stem cells.^136^ In most cases, subpopulations of CSCs have appeared in response to the accumulation of epigenetic and/or genetic alterations in normal stem cells or cancer cells.^137^ It has been reported that cancer cells transfected with OCT3/4, SOX2, KLF4, and c-Myc can be transformed into CSCs.^138^ The presence of CSCs in AML was first confirmed by Bonnet and Dick in 1997.^139^ Subsequently, Al-Hajj et al revealed that the first CSCs observed in solid tumors were found in breast cancer in 2003.^140^ Since then, numerous studies have indicated the presence of CSCs in solid tumors of many tissue types, including breast,^82^ liver,^141^ colorectum,^86^ brain, *etc.*

It is currently well known that the core features of CSCs contain tumorigenicity and self-renewal, as well as drug resistance to chemotherapeutic agents.^137^ Oxygen is an important regulator of cellular metabolism, and HIFs modulate cellular metabolic processes in a hypoxic environment.^137^ As mentioned below, there is growing evidence that HIFs may exert a critical influence on the maintenance and evolution of CSCs. In hypoxia, the activation of HIF-1α not only augmented the number of cluster of differentiation (CD)133-positive glioma stem cells but also potentiated the stemness phenotype of cell lines.^142^ The latter highlighted the expansion of tumor cells that harbor the surface markers CXCR4 (CD184), CD44 (low), and A2B5 in gliomas.^143^ In addition, HIFs induce the self-renewal ability of glioblastoma CSCs and suppress their differentiation.^144^ The effects of hypoxia are mediated by HIF-1α, but not by HIF-2α,^145^ and are closely linked to the activity of NANOG protein in breast cancer stem cells (BCSC).^82^ A key mechanism for BCSC enrichment is the recruitment of NANOG by HIF-1α to synergistically activate transcription of the *TERT* gene, which maintains telomere length and is required for stem cell self-renewal.^82^ Conversely, NANOG itself can also stimulate the transcriptional activity of HIF-1α, serving as positive feedback. ELK3 (Net/SAP-2/Erp), a transcription factor activated by the Ras/extracellular signal-regulated kinase (ERK) signaling pathway, plays an important role in various physiological processes, including cell migration, invasion, angiogenesis, and tumorigenesis.^141^ In 2017, Lee et al^141^ isolated liver cancer stem cells (LCSCs) that expressed CD133 and CD44 from Huh7 HCC cells. They found that ELK3 expression was up-regulated in CD133^+^/CD44^+^ LCSCs compared to non-CD133^+^/CD44^+^ cells; mechanistically, the overexpression of ELK3 increased the metastatic potential of LCSCs by modulating HIF-1α expression.^141^ The activity of HIFs can facilitate the phenotype of stem cells and increase the number of leukemic stem cells (LSCs) in the BM.^146^ In AML, HIF-1α is overexpressed and selectively activated in the CD34^+^/CD38^−^ subpopulation.^147^ In lung cancer, CD133 expression induced by hypoxia was correlated with the binding of OCT4 and SOX2 to the PROM1 promoter.^148^ Meanwhile, another recent study indicated that chronic intermittent hypoxia (CIH) promotes the migration, invasion, and stem cell-like properties of lung cancer cells via the HIF-1α/ATAD2 pathway.^149^ In colorectal cancer cells, the expression of CD44 and OCT4 stem cell markers was reduced upon HIF-1α KD.^150^ The expression of CD24 is highly triggered by hypoxia in human bladder cancer cell lines.^151^ Moreover, co-immunostaining for HIF-1α and CD24 exhibited a statistically significant association in human urothelial carcinoma samples.^151^

### HIF-1α inhibitors and target therapy for tumors

As stated above, HIF-1α is tightly related to angiogenesis, immune escape, as well as the development, metastasis, invasion, and poor prognosis of tumors. Herein, it is critical to further strengthen the relevant studies on HIF-1α and its inhibitors for the understanding and treatment of tumors. To date, several HIF-1α inhibitors are already undergoing clinical trials for oncology treatment, with the underlying mechanisms of action including reduction of HIF-1α transcriptional activity, inhibition of HIF-1α expression, and induction of HIF-1α degradation.

### Reduction of HIF-1α transcriptional activity

Minnelide, a precursor drug to regenerolactone, targets p300 and HSP70 and consequently restricts the transcriptional activity of HIF-1α to treat patients with recurrent pancreatic cancer.^152^ It has been reported that PX-478 could hinder the transcriptional level of HIF-1α and inhibit translation by relying on VHL- and p53-independent mechanisms^153^; the RNA antagonist EZN-2968 inhibited the production of mRNA product for HIF-1α. It is well known that PX-478 and EZN-2968 are dose-dependent drugs that reduce the secretion of HIF-1α and VEGF and cause tumor shrinkage in xenograft animal models, and that satisfactory results were obtained in phase I clinical trials.^154^^,^^155^ Echinomycin, a small molecule antibiotic isolated from a streptomycete, is a potent small molecule inhibitor of HIF-1 that competitively inhibits the activity of HIF-1α by binding specifically to the HRE sequence. In several phases I/II clinical trials in solid tumors, echinomycin has been proven to be toxic and ineffective in the trials and therefore clinical studies were discontinued. Currently, the development of liposomal echinomycin could increase the accessibility and safety of the drug and inhibit the growth and metastasis of tumors.^156^ Hence, a suitable drug formulation could provide a safe and effective therapeutic approach. Topotecan is a topoisomerase inhibitor that can suppress the aggregation of HIF-1α, thereby preventing the ribosomes of HIF-1α mRNA from reaching the binding site to interfere with transcription.^157^ Similarly, YC-1 could inhibit the transcriptional activity of HIF-1α and even the accumulation of proteins.^158^

### Inhibition of HIF-1α expression

EZN-2208 (an irinotecan-modified drug), which can down-regulate the expression of HIF-1α by inhibiting topoisomerase, has been performed in different types of solid tumors; currently, it is being applied in preclinical studies and phase I clinical trials in neuroblastoma.^98^ In preclinical studies, the nanoparticle–drug conjugate CRLX101 at chronic low doses together with BEV reduced BEV-induced up-regulation of HIF-1α and generated synergistic effects with minimal toxicity in mice.^159^ However, it failed to demonstrate its anti-cancer role in early clinical trials^160^ owing to its limited clinical activity. On the other hand, Mo et al^161^ revealed that nickel nanoparticles (Nano-Ni) induce cell malignant transformation via activating the HIF-1α/miR-210/Rad52 pathway, which is involved in DNA damage and DNA repair defect. The utilization of Hsp90 inhibitors (1 μM of 17-AAG, an indirect HIF-1α inhibitor) or KD of HIF-1α attenuated the genotoxic and oncogenic effects of cells by inhibiting the nuclear accumulation of HIF-1α.^161^

### Induction of HIF-1α degradation

As a specific inhibitor of histone methyltransferase G9a, BIX01294 could improve the expression of PHD2 and pVHL (von Hippel-Lindau tumor suppressor protein), thereby promoting the hydroxylation and degradation of HIF-1α, which in turn triggers cell apoptosis and inhibits the proliferation, migration, and invasion of HCC cells.^162^ Additionally, vorinostat, which augments the degradation of HIF-1α with the inhibition of histone dehydrogenase 9, is presently approved by the United States (US) FDA for the treatment of cutaneous T-cell lymphoma.^163^ *In vitro* studies revealed that the HIF-1α inhibitor IDF-11774 inhibited the proliferation, migration, and invasion of tumor cells by promoting the degradation of HIF-1α.^164^ Further investigations are needed to determine its clinical applicability.

## Conclusion and perspectives

In recent years, with the development of cellular immunotherapy and molecular targeted therapy, the therapeutic effect of malignant tumors has markedly improved, but recurrence and drug resistance remains the main cause of death in multiple malignancies. Mechanistically, the adaptation to the HME is a key procedure in the development of most tumors. Hypoxic TME facilitates tumorigenesis, proliferation, and metastasis through underlying mechanisms such as promoting cell proliferation, inhibiting apoptosis, increasing angiogenesis, and boosting cellular immune escape. Indeed, mounting evidence has highlighted the important role of HIF-1α in tumors as the incidence of tumors is increasing, opening a novel avenue for the research of tumor pathogenesis and treatment. It has been observed that HIF-1α is highly expressed in most malignant tumor cells and engaged in inflammatory responses, tumorigenesis, and drug resistance under hypoxic conditions. Currently, a variety of HIF-1α inhibitors have been identified, but all are still at the preclinical or clinical research stage. However, many issues exist in the process of bringing these inhibitors to clinical applications, such as the use of HIF-1α inhibitors as monotherapy or in combination with radiotherapy and chemotherapy, and particularly exploring the efficacy and safety of different HIF-1α inhibitors in conjunction with immunotherapy. Therefore, it is rather essential to deeply understand the mechanism of HIF-1α-induced HME and tumor immune escape, and to find effective translational therapies for the early diagnosis of malignancies and to raise the cure rate as well as to prolong the survival of patients.

## Author contributions

HP: study design. YD and CX: drafting of the manuscript. CY: critical revision of the manuscript. YZ: acquisition, assembly, analysis and interpretation of data, drafting of the manuscript, and critical revision of the manuscript.

## Conflict of interests

The authors declare no conflict of interests.

## Funding

This work was generously supported by the 10.13039/501100001809National Natural Science Foundation of China (No. 82070175), the 10.13039/501100001809Natural Science Foundation of Hunan Province (No. 2022JJ30830) and the Scientific Program of the Health Commission of Hunan Province (China) (No. 20201179).
